# The EspF N-Terminal of Enterohemorrhagic *Escherichia coli* O157:H7 EDL933w Imparts Stronger Toxicity Effects on HT-29 Cells than the C-Terminal

**DOI:** 10.3389/fcimb.2017.00410

**Published:** 2017-09-21

**Authors:** Xiangyu Wang, Yanli Du, Ying Hua, Muqing Fu, Cong Niu, Bao Zhang, Wei Zhao, Qiwei Zhang, Chengsong Wan

**Affiliations:** ^1^Department of Microbiology, School of Public Health, Southern Medical University Guangzhou, China; ^2^Key Laboratory of Tropical Disease Research of Guangdong Province, Southern Medical University Guangzhou, China

**Keywords:** Enterohemorrhagic *Escherichia coli* O157:H7, *espF*, Gene knockout, EspF N-terminal, apoptosis, inflammation

## Abstract

Enterohemorrhagic *Escherichia coli* (EHEC) O157:H7 EspF is an important multifunctional protein that destroys the tight junctions of intestinal epithelial cells and promotes host cell apoptosis. However, its molecular mechanism remains elusive. We knocked out the *espF* sequence (747 bp, Δ*espF*), N-terminal sequence (219 bp, Δ*espF*_*N*_), and C-terminal sequence (528 bp, Δ*espF*_*C*_) separately using the pKD46-mediated λ Red homologous recombination system. Then, we built the corresponding complementation strains, namely, Δ*espF/pespF*, Δ*espF*_*N*_*/pespF*_*N*_, and Δ*espF*_*C*_*/pespF*_*C*_ by overlap PCR, which were used in infecting HT-29 cells and BALB/C mice. The level of reactive oxygen species, cell apoptosis, mitochondrial trans-membrane potential, inflammatory factors, transepithelial electrical resistance (TER), and animal mortality were evaluated by DCFH-DA, double staining of Annexin V-FITC/PI, JC-1 staining, ELISA kit, and a mouse assay. The wild-type (WT), Δ*espF*, Δ*espF/pespF*, Δ*espF*_*C*_, Δ*espF*_*C*_*/pespF*_*C*_, Δ*espF*_*N*_, and Δ*espF*_*N*_*/pespF*_*N*_ groups exhibited apoptotic rates of 68.3, 27.9, 64.9, 65.7, 73.4, 41.3, and 35.3% respectively, and mean TNF-α expression levels of 428 pg/mL, 342, 466, 446, 381, 383, and 374 pg/mL, respectively. In addition, the apoptotic rates and TNF-α levels of the WT, Δ*espF/pespF*, and Δ*espF*_*C*_ were significantly higher than that of Δ*espF*, Δ*espF*_*N*_, Δ*espF*_*C*_*/pespF*_*C*_, and Δ*espF*_*N*_*/pespF*_*N*_ group (*p* < 0.05). The N-terminal of EspF resulted in an increase in the number of apoptotic cells, TNF-α secretion, ROS generation, mitochondria apoptosis, and pathogenicity in BalB/c mice. In conclusion, the N-terminal domain of the Enterohemorrhagic *E. coli* O157:H7 EspF more strongly promotes apoptosis and inflammation than the C-terminal domain.

## Introduction

Enterohemorrhagic *Escherichia coli* (EHEC) O157:H7 is a new foodborne zoonotic pathogen that predominantly colonizes human and animal colorectum. It is transmitted through the fecal-oral route and causes severe diarrhea, hemorrhagic colitis (HC), hemolytic uremic syndrome (HUS), thrombotic thromobocytopenic porpura (TTP), and other gastrointestinal symptoms (Kaper et al., 2004; Rangel et al., 2005).

In 1982, the United States reported the first case of hemorrhagic colitis due to EHEC O157:H7 (Rangel et al., 2005). Since then, similar cases have been occasionally reported in the United States (1993 and 2006), Japan (1996), Canada (2000), China (2000), and other places (Michino et al., 1999; Li et al., 2002; Moist et al., 2010; Heiman et al., 2015). Several EHEC outbreaks have been reported in the past few years. In 2011, 181 people were infected with EHEC due to eating undercooked beef in Japan (Kanayama et al., 2015). In the same year, an EHEC outbreak in Germany was reported, which infected 4,137 people and 52 people died (Frank et al., 2011). In 2014, 119 individuals were infected EHEC O157:H7 via eating infected pork in Canada (Honish et al., 2017). Cattle and swine are the most important intermediate natural carriers of EHEC, as well as the major transmission source to humans (Money et al., 2010; Pennington, 2010). The incidence of human EHEC O157:H7 is positively correlated with the density of cattle (Chase-Topping et al., 2008). The infection of EHEC O157:H7 has strong pathogenicity and lethality, thereby posing a great challenge to global public health.

EspF exists in EHEC, enteropathogenic *Escherichia coli* (EPEC), and *Citrobacter rodentium*, which causes severe disease in humans. The N-terminal of EHEC O157:H7 EspF [length: 1–73 amino acids (aa)] is relatively conserved and the C-terminal (length: 74–248 aa) is composed of four highly homologous proline-rich sequences (McNamara and Donnenberg, 1998; Holmes et al., 2010). However, the molecular pathogenetic role of the N- and C-terminal domains during *Escherichia coli* O157: H7 infection remains unclear.

EspF is a multifunctional effector protein that can destroy the tight junctions of intestinal epithelial cells and promote host cell apoptosis (Viswanathan et al., 2008). Furthermore, the EspF in EHEC O26:H11 and EPEC O127:H6 assists bacteria in avoiding macrophage phagocytosis, although this ability is significantly weaker in EHEC O157:H7 (Tahoun et al., 2011). In 2010, the EspF of EHEC O157:H7 was localized to cell nuclei and determined to disrupt nucleoprotein synthesis and transport within the host cell (Dean et al., 2010). Recently, we confirmed that the N-terminal of EspF targets the mitochondria and induces host cell apoptosis (Zhao et al., 2013). Nevertheless, the molecular pathogenesis of EHEC due to the EspF remains elusive.

Here, we knocked out the EHEC O157:H7 EDL933w *espF* gene and its N- and C-terminal domains by using a λ red homologous recombination system and established its complementation strains. After infection, the level of ROS generation, cell apoptosis, inflammatory cytokines, and animal toxicity were assessed to reveal the role of the EspF in EHEC O157:H7 hemorrhagic enteritis and elucidate the molecular mechanism of EHEC infection and pathogenesis.

## Materials and methods

### Bacteria, plasmids, and cells

Enterohemorrhagic *E. coli* O157:H7 EDL 933(WT), DH5α pKD46, DH5α pKD4, and DH5α were preserved in our laboratory. Prior to infection, the strains were cultured in LB broth at 37°C overnight with 0.1% kanamycin (Δ*espF*, Δ*espF/pespF*, Δ*espF*_*C*_, Δ*espF*_*C*_*/pespF*_*C*_, Δ*espF*_*N*_, and Δ*espF*_*N*_*/pespF*_*N*_) or 0.4% chloramphenicol, 0.1% L- arabinose (Δ*espF/pespF*, Δ*espF*_*C*_*/pespF*_*C*_, and Δ*espF*_*N*_*/pespF*_*N*_). Plasmid pBAD33 has *Xba*l and *Hind*III restriction enzymes sites (ATCC, Manassas, USA) and chloramphenicol-resistant. Its expression was induced by 0.01% L-arabinose. Primers used in this study were synthesized by Sangon Biotech (Shanghai, China) Co., Ltd. Gene sequencing was performed by Guangzhou IGE Biotechnology, Ltd.

HT-29 and Lovo cells were cultured in RPMI-1640 (Gibco, Waltham, USA) broth containing 10% fetal bovine serum (FBS) and 1% penicillin and streptomycin at 5% CO_2_ and 37°C overnight.

Adult female BalB/C mice are obtained from the Lab Animal Center of Southern Medical University [Certificate: SCXK (Guangdong Province) 2016-0041, No.44002100010995]. The mice were housed at the SPF Animal Center of Southern Medical University (Guangzhou, China) according to the regulations of the animal care committee. This study was conducted in accordance with the recommendations of the Southern Medical University Experimental Animal Ethics Committee.

### Construction of EHEC O157:H7 mutant strains

Based on the EHEC O157:H7 *espF* gene sequence (GenBank Accession No. NC_02655), primers H1-K1, H2-K2, H3-K3, and H4-K4 were synthesized to contain homologous arms. The sequences of the primers are shown in Table 1. The 5′ termini of the primers were homologous to the 50-bp upstream and downstream flanking regions of the knocked-out gene. The 3′ termini of the primers were homologous to the end of the kanamycin resistance gene. After PCR amplification, the targeting fragments (with kanamycin resistance) of *espF, espF* N-terminal, and *espF* C-terminal were respectively constructed.

**Table 1:** Sequences of the primers used in this study.

**Primers**	**Sequences (5' → 3')^a^**
H1-K1	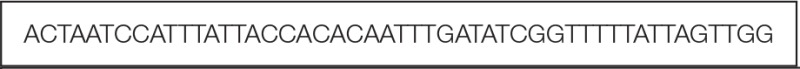 *GTGTAGGCTGGAGCTGCTTC*
H2-K2	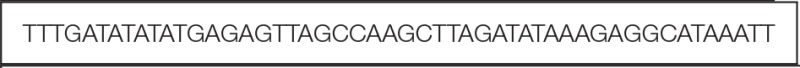 *CATATGAATATCCTCCTTAG*
H3-K3	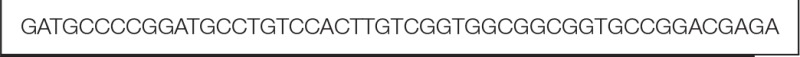 *TGTAGGCTGGAGCTGCTTC*
H4-K4	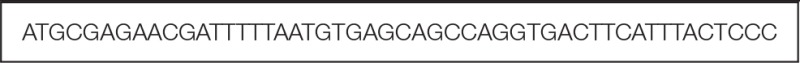 *CATATGAATATCCTCCTTAG*
F1	CCGTTACGACAACACCTC
F2	CGGTGCCCTGAATGAACTGC
R1	GGATTCATCGACTGTGGCCG
R2	TACCCAGCCACTACCATT
pkdF	GGAGCGCATGGCAGAACAC
pkdR	CAGAGCGGCAATAAGTCG
pF	ATGCCATAGCATTTTTATCC
pR	GATTTAATCTGTATCAGG
eF	CCCAAGCTTTTACCCTTTCTTCGATTGCT
eR	CTGTCTAGA**AGGAGG**AATTCACCATGCTTAATG GAATTAGTAA
NF	CCCAAGCTTTTAGGGAGTAAATGAAGTCACCTG
CR	CTCTAGA**AGGAGG**AATTCACCATGTCTCGTCCG GCACCGCCGCC

a *The sequence in the box represents the homologous arm of the espF gene. The italicized letters represent the homologous arms of kana gene. The underlined sequence TCTAGA represents the restriction sites of XbaI; AAGCTT indicates the restriction of HindIII; Bold letters represent synthetic ribosomal binding sites*.

**Figure 1:**
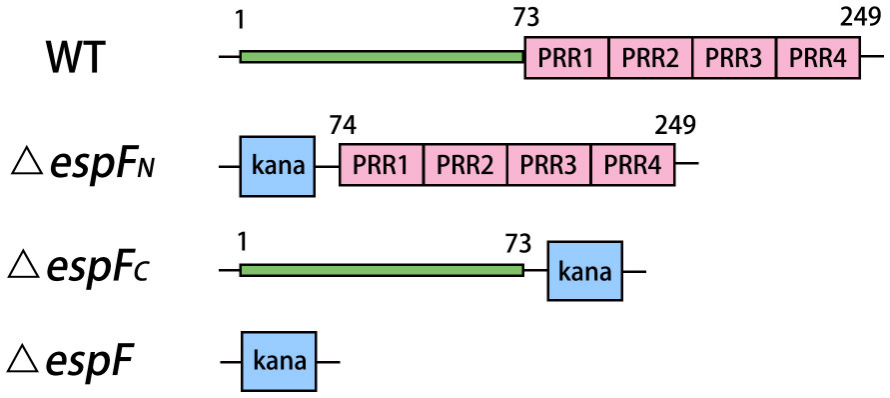
Modular architecture of *espF* mutant strains. Deletion sites of the mutant strains are replaced by kanamycin resistance gene. The N-terminal of EspF region are residues 1–73. And C-terminal of EspF region are residues 74–249.

Plasmid pKD46 was transformed into EHEC O157:H7 EDL933 and cultured to an OD_600_ = 0.2–0.3. The recombinant enzyme Exo, Bet, and Gam of pKD46 were fully expressed after adding 10–30 mmol/L L-arabinose. Approximately 10 μL of the targeting fragments from the gels were transferred into a gene pulser cuvette (Bio-Rad Laboratories, Inc.) and subjected to an electric shock for 10–20 s (25 μF, 200Ω, 3KV). The transfected bacteria were grown in LB plates (containing 100 μg/mL kanamycin) at 37°C overnight, and positive colonies were selected for PCR analysis and gene sequencing verification. The deletion sites of the mutant strains are presented in Figure 1.

### Construction of EHEC O157:H7 complementation strains

The primers EF, ER, N-F, C-R, pF, and pR were designed based on the sequence of EHEC *espF* and plasmid pBAD33 (Table 1). Using primers eF, eR, N-F, and C-R, and the *espF* gene (779 bp) and its N-terminal (252 bp), the C-terminal (561 bp) was amplified by overlap-PCR (OL-PCR). The PCR conditions were as follows: 95°C for 5 min; followed by 29 cycles of 94°C for 40 s, 56°C for 30 s, and 72°C for 50 s; and a final extension of 72°C for 5 min. The targeting fragment and vector pBAD33 were digested by restriction enzymes (*Xba*I and *Hind*III) and connected by using a T4 DNA ligase at 4°C for 12 h. The recombinant plasmids were transformed into Δ*espF*, Δ*espF*_*N*_, and Δ*espF*_*C*_ competent strains by electric shock (25μF, 200Ω, 3 KV for 10–20 s). Monoclonal colonies were selected and confirmed by cross-PCR. Positive colonies were selected for gene sequencing verification.

Total RNA of the collected complementation strains was extracted using a bacterial RNA kit (Omega, Norcross, USA). DNase I digestion (Takara, Japan) was performed to remove the any contaminating DNA, and a Prime Script™ II 1st strand cDNA synthesis kit (Takara) was employed to reversely transcribe the RNA into cDNA. Using primers E-F, E-R, N-F, and C-R, RT-PCR was conducted using the cDNA templates to confirm whether the complementation genes were successfully expressed. The RT-PCR conditions were as follows: 95°C 3 min; followed by 30 cycles of 95°C for 30 s, 55°C for 30 s, and 72°C for 50 s; and a final extension step of 72°C for 5 min.

### Reactive oxygen species (ROS) assay

Before infection, the strains of different groups were cultured overnight in 5 mL of LB broth at 37°C. The HT-29 cells were cultured in 12-well cell culture plates (about 5 × 10^5^ cells per well) overnight. These were infected with the wild-type (WT), Δ*espF*, Δ*espF*_*N*_, Δ*espF*_*C*_, *pespF, pespF*_*C*_, and *pespF*_*N*_ at a 1:100 rate and incubated at 37°C in 5% CO_2_ for 6 h. Then, the cells were washed with phosphate-buffered saline (PBS, 0.01 mol/L, pH 7.4) three times and then collected by trypsin digestion. An ROS assay kit (Beyotime Biotechnology, Jiangsu, China) was used to detect ROS levels within the host cells. DCFH-DA was added to the cells at a final concentration of 10 μM and incubated at 37°C under moist dark conditions for 20 min. The results were measured by using a fluorescence microplate reader (Infinite M200, TECAN) at an excitation wavelength of 485 nm and an emission wavelength of 525 nm. All determinations were performed in triplicate.

### Cell apoptosis assay

The HT-29 cells were infected with EHEC O157:H7 WT, Δ*espF*, Δ*espF*_*N*_, Δ*espF*_*C*_, *pespF, pespF*_*C*_, and *pespF*_*N*_ and incubated at 37°C in 5% CO_2_ for 6 h. Then, the cells were washed and collected at a density of 1 × 10^6^ cells per tube. An annexin V-FITC apoptosis detection kit (Keygen Biotech, Nanjing, China) was used to detect apoptotic cells. Annexin V-FITC and propidium iodide (PI) were added to the collected cells for 15 min at room temperature in the dark. The cells were analyzed immediately by flow cytometry (BD FACSCalibur, USA). Annexin-V-FITC was excited at a wavelength of 488 nm, and fluorescence emission was detected using a 530-nm band-pass filter. PI was excited at a wavelength of 488 nm, and emission was detected using a 630-nm band-pass filter.

The HT-29 cells were infected with EHEC O157:H7 WT, Δ*espF, pespF*, Δ*espF*_*N*_, and Δ*espF*_*C*_ and incubated at 37°C in 5% CO_2_ for 6 h. The cleavage products of the chromogenic caspase substrates, Ac-DEVD-pNA and Ac-LEHD-pNA, were separately measured to quantify the activity of caspase-3 and caspase-9 using an assay kit (Beyotime Biotechnology, Jiangsu, China). The HT-29 cells were collected and lysed on ice for 15 min. Then, the protein was collected by centrifugation at 16,000 *g* for 20 min at 4°C. Approximately 50–100 μg protein was added to a reaction buffer containing Ac-DEVD-pNA or Ac-LEHD-pNA (2 mM), incubated at 37°C for 10 h, and the absorbance of yellow pNA (the cleavage product) was calculated using a microplate reader (Infinite M200, TECAN) at a wavelength of 405 nm. The specific activity of caspase-3 and caspase-9 was normalized to the 50 ug protein in the HT-29 cell per well.

### JC-1 assay

The HT-29 cells were cultured in six-well cell culture plates (about 1 × 10^6^ cells per well) at 37°C overnight and infected with EHEC WT, Δ*espF*, Δ*espF*_*N*_, Δ*espF*_*C*_, *pespF, pespF*_*C*_, and *pespF*_*N*_ for 2 h. A JC-1 apoptosis detection kit (Keygen) was used to measure the mitochondrial transmembrane potential (Δψm). After washing, the JC-1 working solution was added to each well, followed by incubation at 5% CO_2_ and 37°C for 20 min. The results were observed under a fluorescence microscope (TE2000-U, Nikon).

After infection, the cells were washed and collected by trypsin digestion. The cells were stained with JC-1 for 20 min in the dark. Then, the cells were washed twice and resuspended in 500 μL of an incubation buffer. The cells were immediately analyzed by flow cytometry (BD FACSCalibur, USA) using a 488-nm excitation wavelength with a 530-nm band-pass emission filter through FL1 an FL2 tunnel.

### Measurement of transepithelial electrical resistance (TER)

The HT-29 cells were seeded at a density of 10^5^ cells/cm^2^ onto 0.4-μm pore size, 1.13 cm^2^ surface area polyester Transwell® cell culture inserts (Corning-Costar, NY) and infected with EHEC WT, Δ*espF, pespF*, Δ*espF*_*N*_, and Δ*espF*_*C*_ for 6 h. TER was measured after 6 h using an EVOM^2^ epithelial voltohmmeter (World Precision Instruments, Sarasota). TER values, which were reported as means ± SE, were obtained from three measurements per condition.

### Detection of inflammatory cytokines

The HT-29 cells were cultured in 24-well cell culture plates (1 × 10^5^ cells per well) at 37°C overnight and infected with EHEC WT, Δ*espF*, Δ*espF*_*N*_, Δ*espF*_*C*_, *pespF, pespF*_*C*_, and *pespF*_*N*_ for 6 h. The cell culture supernatants were then centrifuged for 15 min at 1,000 *g* at 4°C to remove particulates. Tumor necrosis factor α(TNF-α) levels were measured using a specific ELISA kit (Cusabio, Wuhan, China). The process was as follows: Approximately 100μL of the standard or sample was added into each well and incubated at 37°C for 2 h. After incubation, the supernatant was removed, and 100μL of a biotin antibody was added, and then incubated at 37°C for 1 h. After incubation, the plates were washed thrice, and the 100μL of HRP-avidin was added and incubated at 37°C for 1 h. After incubation, the plates were washed five times. Then, 90 μL of the TMB substrate was added to each well, followed by incubation at 37°C for 15–30 min in the dark. Finally, 50 μL of a stop solution was added to each well, and the results were evaluated within 5 min using a microplate reader at a wavelength of 450 nm.

### Survival assay of mice

A total of 50 adult female BalB/C mice (5–6 weeks old, weight: 14.29 ± 1.12 g) were randomly divided into eight groups (WT, Δ*espF*, Δ*espF*_*N*_, Δ*espF*_*C*_, and uninfected group), with 10 mice in every group. The EHEC O157 of the different groups was cultured overnight in kanamycin, chloramphenicol, or L-arabinose at 37°C. The mice respectively received 0.3 mL of the bacterial suspension of different groups (approximately 2 × 10^10^ CFU/mL) by intragastric administration. After 12 h, the mice were infected again with 0.3 mL of the bacteria suspension (approximately 1.5 × 10^10^ CFU/mL). The uninfected group received LB broth. Prior to the gavage, the BalB/C mice were injected with mitomycin C (2.5 mg/kg) to enhance susceptibility to bacteria. The survival state of the mice was monitored every 4 h for 15 days, and their survival rate was calculated. After 15 days, the surviving mice were killed and their colons (5.5 cm of distal colon) were taken out. The fecal particles inside the colons were gently removed. Subsequently the weight of each colon was measured.

### Statistical analysis

All experiments, unless otherwise stated, were performed independently in triplicate. The data were expressed as the mean ± SD of triplicate experiments. When necessary, one-way ANOVA was performed, with *p*-values < 0.05 signifying statistical significance.

## Results

### Construction of EHEC O157:H7 Δ*espF*, Δ*espF_*N*_*, and Δ*espF_C_* mutant strains and their complementation strains

**Figure 2:**
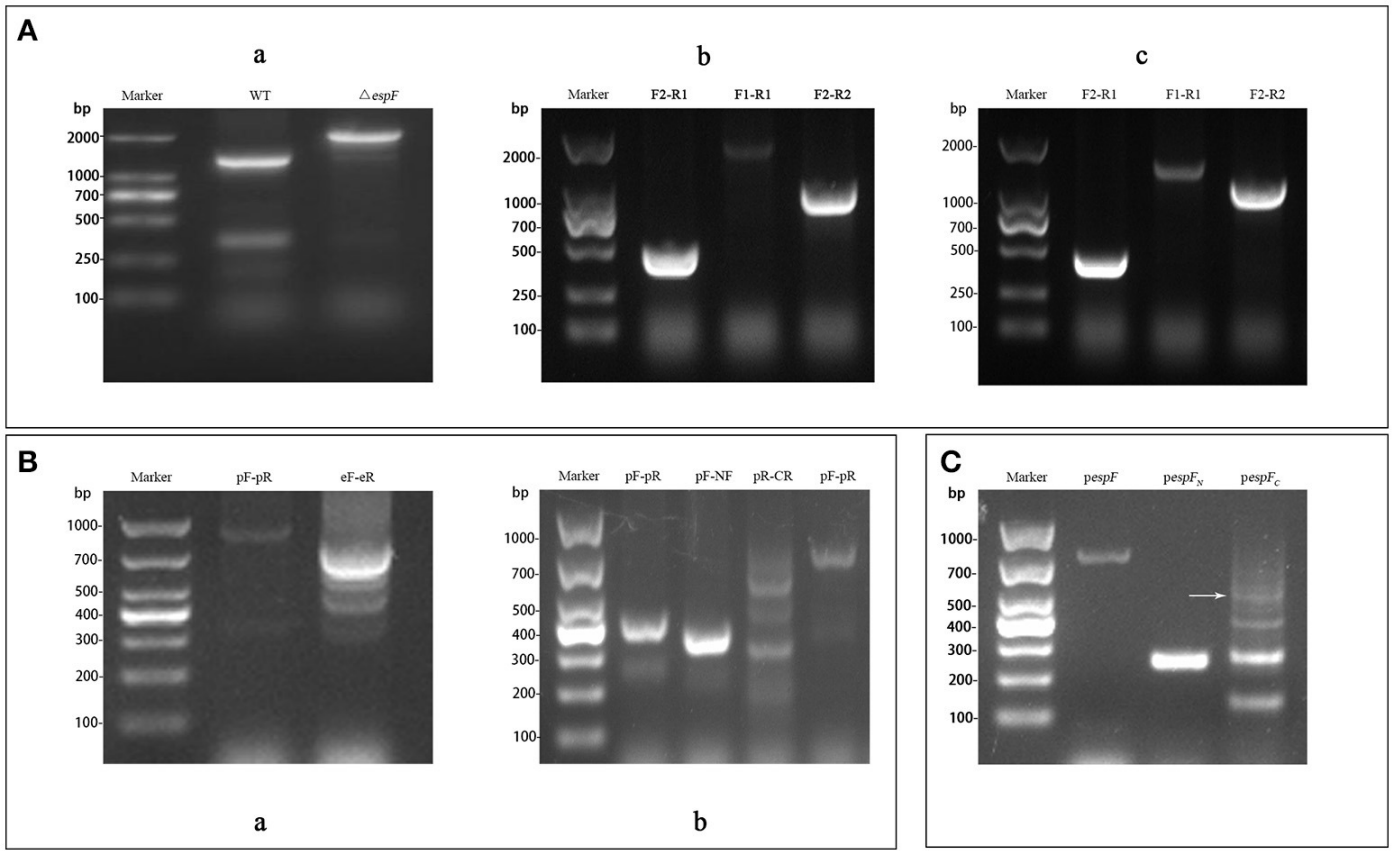
Construction of the EHEC O157:H7 mutant and complementation strains. **(A)** Electrophoresis verification of the mutant strains. **(a)** WT: Amplification of wild-type using primers F1-R2 (1,289 bp); Δ*espF***:** Amplification of Δ*espF* using primers F1-R2 (2,019 bp); **(b)** F2-R1: Amplification of Δ*espF*_*N*_ using primers F2-R1 (471 bp); F1-R1: Amplification of Δ*espF*_*N*_ using primers F1-R1 (2,015 bp); F2-R2: Amplification of Δ*espF*_*N*_ using primers F2-R2 (1,002 bp); **(c)** F2-R1: Amplification of Δ*espF*_*C*_ using primers F2-R1 (471 bp); F1-R1: Amplification of Δ*espF*_*C*_ using primers F1-R1 (1,487 bp); F2-R2: Amplification of Δ*espF*_*C*_ using primers F2-R2 (1,221 bp). **(B)** Identification of complementation strains by cross PCR. **(a)** pF-pR: Amplification of *pespF* using primer pF and pR (948 bp); eF-eR: Amplification of *pespF* using primers eF and eR (779 bp); **(b)** pF-pR: Amplification of *pespF*_*N*_ using primers pF and pR (423 bp); pF-NF: Amplification of *pespF*_*N*_ using primer pF and NF (373 bp); pR-CR: Amplification of *pespF*_*C*_ using primers pR and CR (607 bp, white arrow); pF-pR: Amplification of *pespF*_*C*_ using primers pF and pR (732 bp). **(C)** Agarose gel electrophoresis of the RT-PCR products. *pespF*: RT-PCR results of *pespF* (779 bp); *pespF*_*N*_: RT- PCR results of *pespF*_*N*_ (252 bp); *pespF*_*C*_: RT-PCR results of *pespF*_*C*_ (557 bp, arrow).

H1K1 and H2K2, H3K3 and H2K2, and H1K1 and H4K4 were respectively used as primers for PCR amplification of the targeting fragment (with kanamycin resistance) of Δ*espF*, Δ*espF*_*N*_, and Δ*espF*_*C*_. Targeting fragments were electrostatically transformed into cells of the EHEC O157:H7 strain, and the *espF, espF-N*, and *espF-C* genes were knocked out by λ red homologous recombination. Cross-PCR was performed to verify each mutant strain by using internal primer F2R1 and external primer F1R2. The bands of the mutant strains were separated via electrophoresis. The resulting band of Δ*espF* by using primer F1R2 was 2,019 bp in size (Figure 2Aa, containing kanamycin). The resulting band of Δ*espF*_*N*_ using primer F1R1 was 2,015 bp in size (Figure 2Ab, containing kanamycin and *espF-C* gene). The resulting band of Δ*espF*_*C*_ using primer F2R2 was 1,221 bp in length (Figure 2Ac, containing kanamycin and *espF-N* gene). The target bands coincided with the theoretical values. Sequencing indicated that we successfully generated the EHEC O157:H7 mutant strains.

Using the whole genome of EHEC O157:H7 as template, the gene fragments of *espF* (779 bp) and its N-terminal (252 bp) and the C-terminal (561 bp) were respectively amplified via OL-PCR (overlap PCR). The targeting fragments were ligated to the pBAD33 plasmid, which were then transformed into the corresponding mutant strains. Then, the band position of complementation strains was analyzed by electrophoresis. The respective sizes of the bands of *pespF, pespF*_*N*_, and *pespF*_*C*_ using the external primer pF-pR were 948 bp (Figure 2Ba, containing the pBAD33 fragment), 423 bp (Figure 2Bb), 732 bp (Figure 2Bb). The target bands coincided with our theoretical values. Sequencing verified that we had successfully generated the EHEC O157:H7 complementation strains.

The complementation strains were induced by L-arabinose and collected after growth up to the logarithmic phase. Total RNA was extracted from the strains and reversed transcribed into cDNA for the subsequent RT-PCR analysis. The results of RT-PCR electrophoresis were as follows: 779 bp (*pespF*), 252 bp (*pespF*_*N*_), and 557 bp (*pespF*_*C*_). The size of the gene fragments was consistent with the theoretical values (Figure 2C, containing the enzyme cutting sites and ribosomal binding sites). These results proved that we had successfully constructed the complementation strains.

### The Δ*espF* decreases ROS production in HT-29 cells

**Figure 3:**
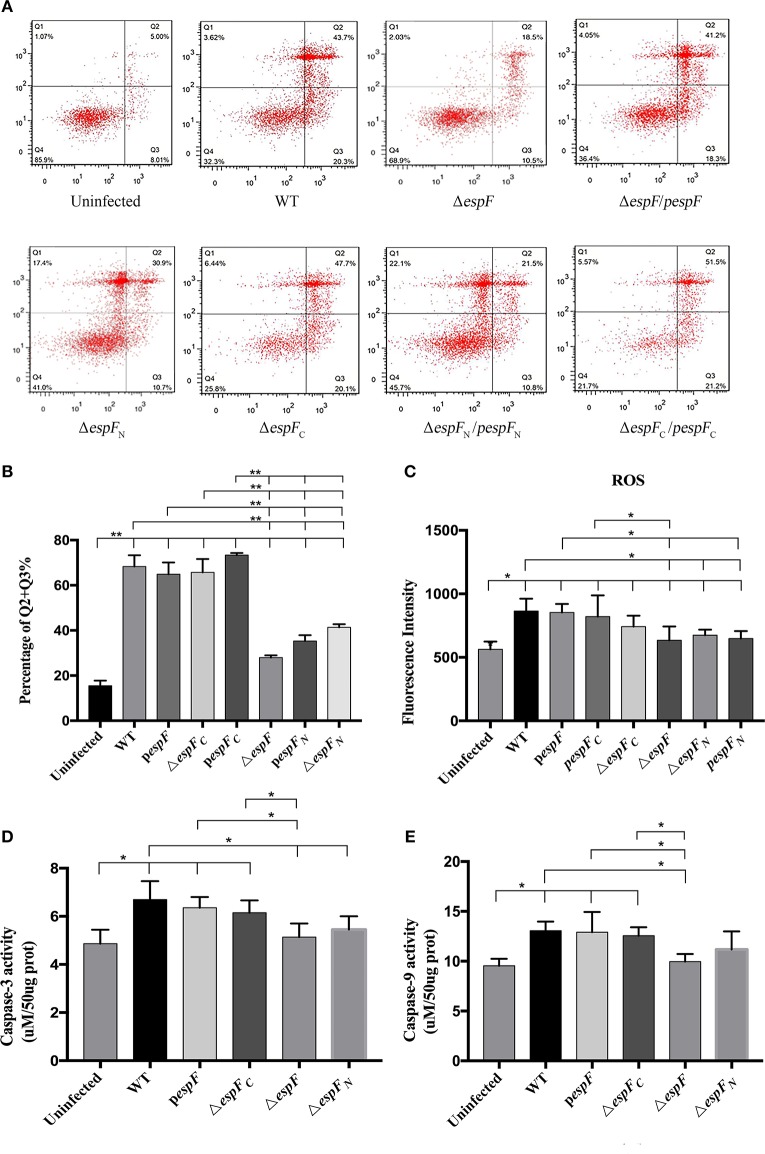
Apoptosis of HT-29 cells after infection. **(A)** Detection of HT-29 apoptotic cells after infection as detected by double-staining using annexin V-FITC and PI combined with flow cytometry. Q1 represents the dead cells (Annexin V-FITC-/PI+); Q2 represents the late apoptotic cells (Annexin V-FITC+/PI+); Q3 represents the early apoptotic cells (Annexin V-FITC+/PI−); Q4 represents the viable cells (Annexin V-FITC-/PI-). **(B)** The proportion of early and late apoptotic cells in each experimental group (Q2+Q3%). Data are expressed as the mean ± SD of at least three independent experiments. **(C)** The reactive oxygen species (ROS) levels of the HT-29 cells after infection. The fluorescence intensity of each group (^*^p < 0.05) using an excitation wavelength of 488 nm and emission wavelength of 525 nm. **(D,E)** Detection of caspase-3 and 9 activity. Cells were treated by different strains, and then caspase-3 and 9 activity were examined. Data are expressed as the mean ± SD of at least four independent experiments.

After Δ*espF, pespF*, and WT infection of the HT-29 cells, ROS levels were measured using the DCFH method. The mean green fluorescence values of Uninfected, WT, Δ*espF, pespF*, Δ*espF*_*C*_, *pespF*_*C*_, Δ*espF*_*N*_, and *pespF*_*N*_ was 394, 864, 635, 853,742, 820, 675, and 649, respectively (Figure 3C). The green fluorescence intensity of each group was higher than that of the uninfected group (*P* < 0.05). The fluorescence intensity of WT was higher than those of Δ*espF*, Δ*espF*_*N*_, and *pespF*_*N*_ (*P* < 0.05). The fluorescence intensity of *pespF* was higher than those of Δ*espF* and *pespF*_*N*_ (*P* < 0.05). In addition, the fluorescence intensity of *pespF*_*C*_ was higher than that of Δ*espF*. Other groups showed no significant differences. These findings demonstrated that the ROS levels of WT, *pespF*, and *pespF*_*C*_ were higher than those of Δ*espF*, Δ*espF*_*N*_, and *pespF*_*N*_. It indicated that the deletion of the *E. coli* O157:H7 *espF* gene and its N-terminal led to a decrease in ROS levels in the host cells.

### The N-terminal domain of EspF plays an important role in apoptosis

Apoptosis in the infected HT-29 cells was assessed by double staining of Annexin V-FITC/PI combined with flow cytometry (Figure 3A). The average apoptotic percentage (early and late apoptosis) of the WT, Δ*espF, pespF*, Δ*espF*_*C*_, *pespF*_*C*_, Δ*espF*_*N*_, and *pespF*_*N*_ was 68.3, 27.9, 64.9, 65.7, 73.4, 41.3, and 35.3%, respectively (Figure 3B). Compared to the uninfected group (13.1%), the number of early and late apoptotic cells significantly increased in each experimental group (*P* < 0.01). Compared to the Δ*espF*, Δ*espF*_*N*_, and *pespF*_*N*_ groups, the *pespF*, Δ*espF*_*C*_, and *pespF*_*C*_ groups showed fewer viable cells and a significantly higher number of apoptotic cells (*P* < 0.01). The activity of caspase-3 and caspase-9, two markers of apoptosis, were also detected to further assess the apoptotic effect of EspF. As indicated in Figures 3D,E, treatment with WT, *pespF* or Δ*espF*_*C*_ led to a remarkable activation of caspase-3 and caspase-9 (*P* < 0.05), which was significantly higher than uninfected and Δ*espF* group. Furthermore, caspase-3/9 activation of Δ*espF*_*N*_ reduced.

Thus we found that the apoptotic rate of Δ*espF*_*C*_ and *pespF*_*C*_ remained high after mutation, indicating that the deletion of the C-terminal gene did not reduce the ability of strains to induce host cell apoptosis. On the other hand, the apoptotic rate of Δ*espF*_*N*_ significantly decreased. These findings revealed that EspF plays an important role in promoting apoptosis in the host cells, and its N-terminal domain is indispensable, which were in agreement with our previous results.

### The *espF*-mutant strains exhibit a slower rate of reduction in Δψm

**Figure 4:**
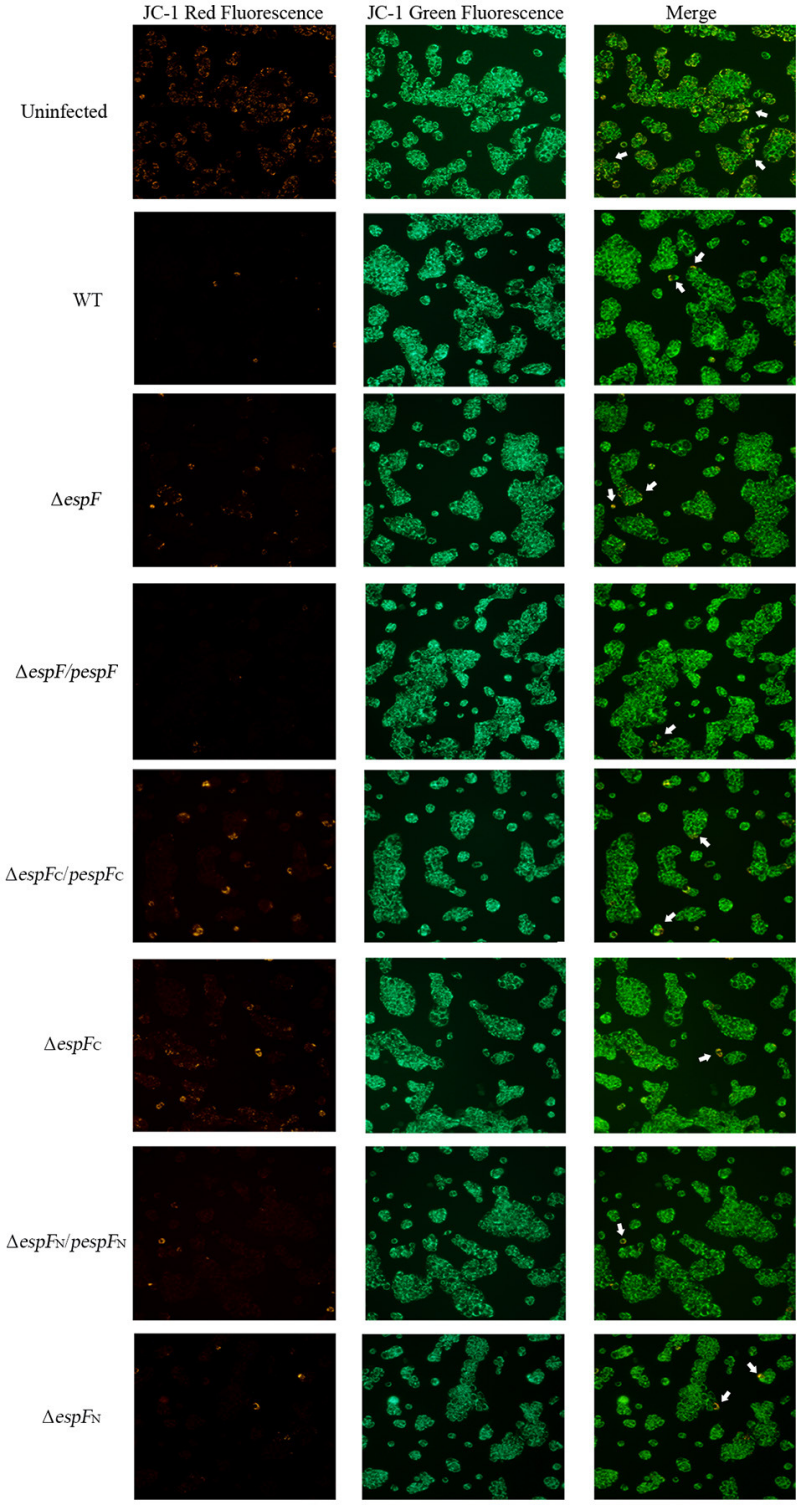
Changes in the mitochondrial transmembrane potential of HT-29 cells. The red fluorescence (polymer) and green fluorescence (monomer) of HT-29 cells after infection. Normal mitochondria JC-1 forms a polymer using its potential energy, which is emitted as red fluorescence. During disruption of mitochondrial function, JC-1 is dispersed as monomers and distributed in the cytoplasm, which is detected as green fluorescence. Merge: The overlay of red fluorescence (polymer) and green fluorescence (monomer) in HT-29 cells after infection. White arrows point to the normal cells which have strong red and green fluorescence.

After infection, the mitochondrial Δψm of the HT-29 cells was detected by JC-1 staining (Figure 4). Strong green fluorescent signals and sporadic red fluorescent signals were observed in the WT, *pespF*, Δ*espF*_*C*_, and *pespF*_*C*_ groups, indicating a significant decrease in mitochondrial Δψm, which in turn results in mitochondrial dysfunction and eventually mitochondrial apoptosis. The Δ*espF, pespF*_*N*_, and Δ*espF*_*N*_ groups showed strong green fluorescent signals and weak red fluorescent signals. The fluorescence intensity of the red signals was significantly higher than that of the above groups, but lower than that of the control group (strong green signals and red fluorescent signals). These also exhibited a decrease in Δψm but not as severe as that in the WT, *pespF*, Δ*espF*_*C*_, and *pespF*_*C*_.

**Figure 5:**
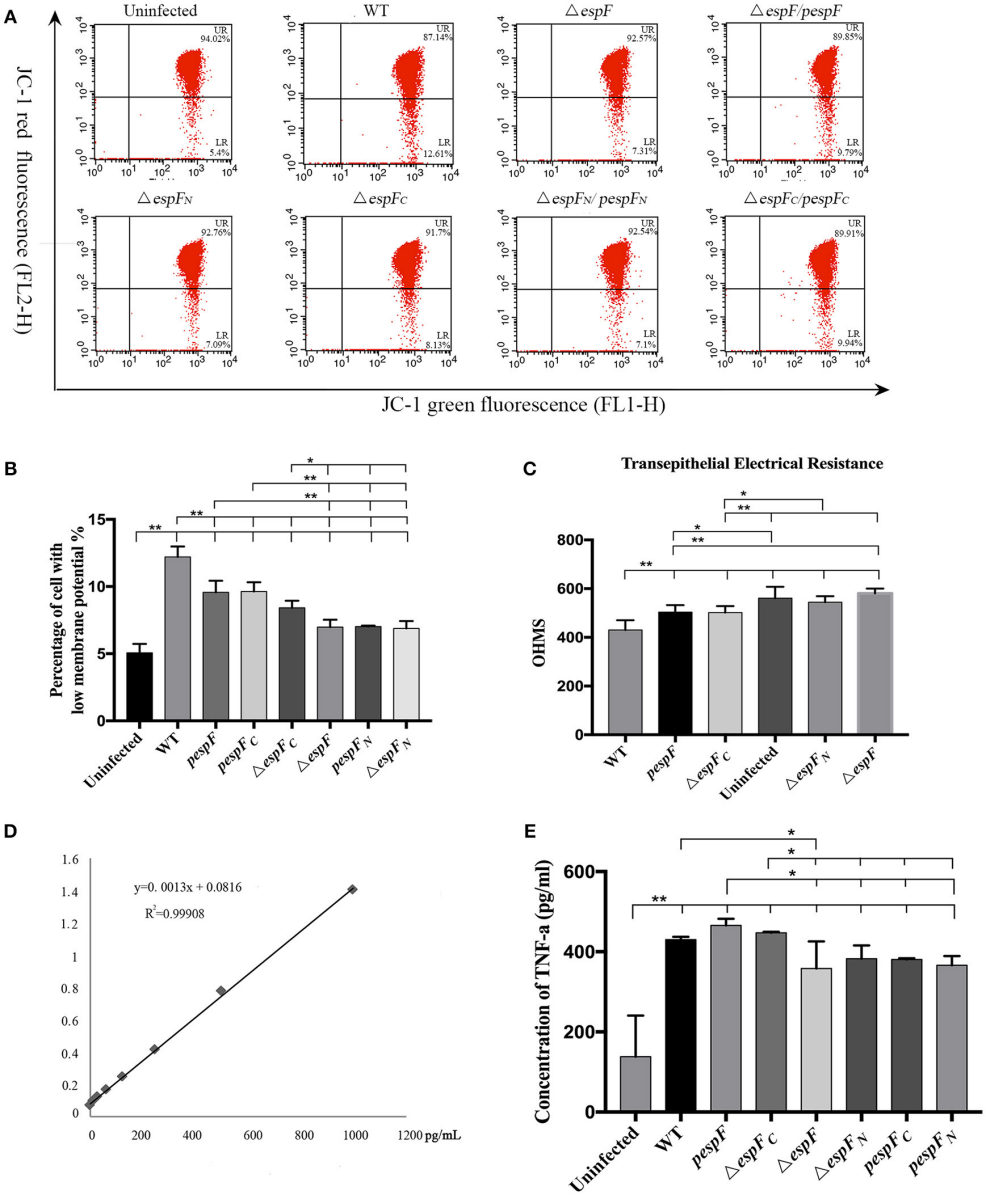
**(A)** Flow cytometry analysis of changes in the mitochondrial transmembrane potential. Flow cytometry analysis was performed to assess mitochondrial membrane potential (MMP). The cells with weak red fluorescence indicated lower membrane potential (LR quadrant). The numbers at the corners displayed the percentage of cells in the corresponding quadrants. **(B)** The percentage of cells with low MMP (LR) values in each group is shown. Data are expressed as the mean ± SD of at least three independent experiments. **(C)** Measurement of transepithelial electrical resistance (TER) after 6 h incubation. (^*^p < 0.05; ^**^p < 0.01) **(D)** TNF-α expression in HT-29 cells after infection. Standard curve of TNF-α. **(E)** The level of TNF-α secreted by HT-29 cells after infecting each group.

The cells were also subjected to flow cytometry analysis (Figure 5A). The percentage of cells exhibiting low membrane potential (strong green fluorescence intensity and weak red fluorescence intensity) in the Uninfected, WT, Δ*espF, pespF*, Δ*espF*_*N*_, Δ*espF*_*C*_, *pespF*_*N*_, and *pespF*_*C*_ groups was 5.1, 12.2, 7.0, 9.6, 6.9, 8.4, 7.0, and 9.6% (Figure 5B). The Δψm of the WT, *pespF, pespF*_*C*_, and Δ*espF*_*C*_ groups was lower than that of the Δ*espF*, Δ*espF*_*N*_, and *pespF*_*N*_ groups (*p* < 0.01).

Above all, these findings demonstrated that the EHEC O157:H7 wild strain, *pespF*, and Δ*espF*_*C*_ could reduce the Δψm in the host cells, and the Δ*espF* and Δ*espF*_*N*_ strains slow down the process of Δψm reduction.

### The N-terminal of *espF* is involved in tight junction disruption

After 6 h of incubation, a progressive decrease in TER was observed in HT-29 cells infected with WT, Δ*espF*_*C*_ (*p* < 0.01), and *pespF* (*p* < 0.05) but not Δ*espF* and Δ*espF*_*N*_, indicating a loss in barrier function (Figure 5C). Interestingly, Δ*espF*_*C*_ was equally efficient in complementing this phenotype, thereby suggesting that the N-terminal of *espF* is involved in the disruption of tight junctions.

### Infection with *espF*-mutant strains decrease TNF-α secretion in HT-29 cells

**Figure 6:**
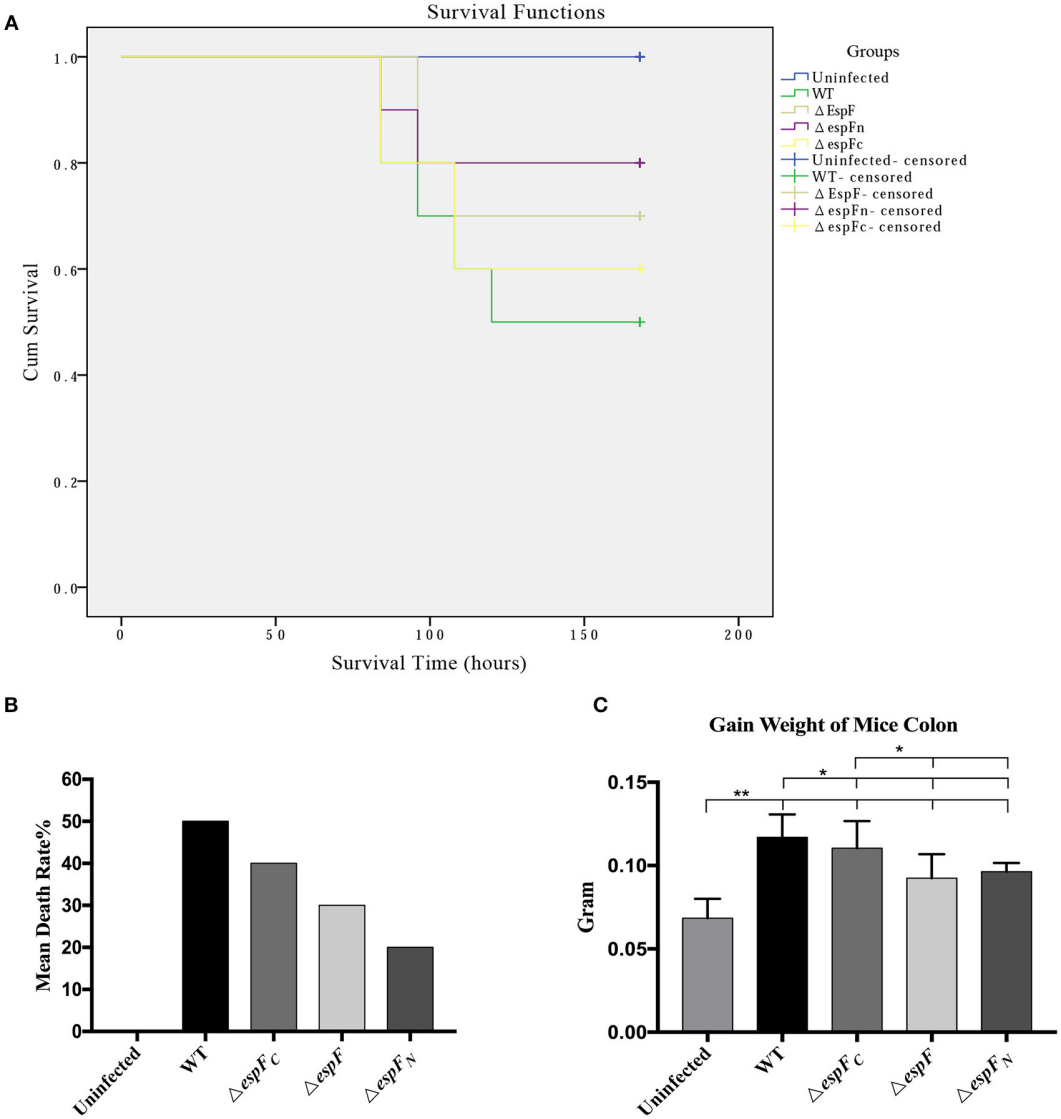
Pathogenicity of EHEC O157:H7 mutant strains to BALB/c mice. **(A)** Survival curves of BALB/c mice post gavage of the constructed strains. Being censored means mice were still alive after 15 days. **(B)** The mean mortality of mice after infecting the constructed strains. **(C)** Increase in colon weight of the mice at 15 days post infection. (^*^p < 0.05; ^**^p < 0.01).

The standard curve of TNF-α was successfully established in Figure 6A. The concentration of TNF-α secreted by HT-29 cells respectively infected with WT, pΔ*espF*, Δ*espF*_*C*_, Δ *espF*, Δ*espF*_*N*_, pΔ*espF*_*C*_, and pΔ*espF*_*N*_ was 428, 466, 446, 342, 383, 381, and 374 pg/mL (Figure 6B). Compared to the control group (113 pg/mL), the TNF-α level of each experimental group significantly increased (*p* < 0.01), with that of *pespF* and Δ*espF*_*C*_ higher than that of Δ*espF*, Δ*espF*_*N*_, *pespF*_*C*_, and *pespF*_*N*_ (*p* < 0.05). In addition, the TNF-α level of the WT group was higher than that of Δ*espF* (*p* < 0.05).

The deletion of the *espF* gene resulted in a reduction in TNF-α secretion in HT-29 cells. The concentration of TNF-α of the *pespF*_*C*_ and *pespF* groups did not significantly differ from that of the WT but was higher than that of the other mutant strains. The deletion of the *espF* gene resulted in a decrease in TNF-α expression in the host cells. These findings indicate that the N-terminal domain of EspF plays an important role in the infection process.

### The *espF*-mutant strains attenuate pathogenicity to BalB/c mice

After intraperitoneal injection of mitomycin and two rounds of gavage of the constructed strains, the BalB/c mice were monitored each day. The mortality rates of the WT, Δ*espF*, Δ*espF*_*N*_, Δ*espF*_*C*_, and uninfected groups were 50, 30, 20, 30, and 0% (Figure 6B). The survival curve is presented in Figure 6A.

The WT mice showed the highest mortality rate (50%), whereas that of the Δ*espF* (30%) and Δ*espF*_*N*_ (20%) mice were low. The mortality rate of the Δ*espF*_*C*_ mice was relatively high (40%), although these also died early. After 15 days, the surviving mice were killed and their colons (5.5 cm of the distal colon) were weighed. The mean colon weight of the WT, Δ*espF*, Δ*espF*_*N*_, Δ*espF*_*C*_, and uninfected groups were 0.117, 0.0924, 0.0963, 0.1104, and 0.06846 g, respectively (Figure 6C). The distal colon of the WT and Δ*espF*_*C*_ groups was significantly heavier than that pf the Δ*espF* and Δ*espF*_*N*_ groups (*p* < 0.05). After infection, the colons of the mice showed signs of proliferation, inflammation, edema, and increase in weight. These results indicate that the WT and Δ*espF*_*C*_ groups developed more severe pathological reactions than the other groups. These findings reveal that the deletion of the *espF* gene attenuates the pathogenicity of EHEC O157:H7 to BalB/c mice, particularly the N-terminal domain.

## Discussion

Amino acids (aa) 1–24 of the EspF comprise the mitochondrial targeting signal (MTS) (Nougayrede and Donnenberg, 2004), whereas aa 21–74 represent nucleolar targeting domain (NTD) (Dean et al., 2010). The EspF of *Citrobacter rodentium* is capable of initiating cell apoptosis (Nagai et al., 2005). In 2013, our group knocked out aa 15–68 of EspF (EHEC O157:H7) and compared the adhesion and toxicity between the WT and Δ*espF*. We verified that the N-terminal of EspF could migrate and target the host mitochondria, increase membrane permeability, activate caspase-3 signaling pathways, change the potential of mitochondrial membrane (Δψ), and finally induce cell apoptosis. However, whether the C-terminal of EspF (including the four PxxP domains) induces cell apoptosis was unclear. The expression of the host proteins was altered during infection. Furthermore, our understanding of the mechanism underlying the regulation of host cell activities by EspF remained unclear.

In the present study, we successfully knocked out the entire *espF* gene (1–249 aa), the N-terminal domain (1–73 aa), and the C-terminal domain (74–249 aa) and constructed EHEC O157:H7 mutant strains (Δ*espF*, Δ*espF*_*N*_, and Δ*espF*_*C*_). We inserted the transformed pBAD33 plasmid (containing target fragment, constructed by OL-PCR) into mutant strains and constructed complementation strains (Δ*espF/pespF*, Δ*espF*_*N*_*/pespF*_*N*_, and Δ*espF*_*C*_*/pespF*_*C*_). The HT-29 cells were infected by WT, mutant strains, and complementation strains, followed by detection of cell apoptosis, ROS, inflammatory reaction, mitochondrial apoptosis, and mice assay. The results of these tests provide insights into the function of the N- and C-terminal domains of EspF.

Mitochondria are the “master switches” that regulate cell apoptosis. When cells are stimulated by death signals, the membrane permeability of the mitochondria increases and apoptosis-related factors are released, which in turn triggers a cascade of reactions that induce cell apoptosis(Sinha et al., 2013). The MTS generally consists of the first 101 amino acids of the EspF sequence. Mitochondrial targeting is disrupted when the16th aa of the MTS is mutated from leucine to glutamic acid (Nagai et al., 2005).

The N-terminal of EspF targets the mitochondria and induces cell apoptosis (Nougayrede and Donnenberg, 2004). By JC-1 staining, WT, *pespF*, Δ*espF*_*C*_, and *pespF*_*C*_ showed strong green and sporadic red fluorescent signals; Δ*espF, pespF*_*N*_, and Δ*espF*_*N*_ exhibited strong green and weak red fluorescent signals; and the uninfected cells presented strong green and strong red fluorescent signals. These findings are indicative of host mitochondrial dysfunction (a decrease in mitochondrial Δψm) with the WT, *pespF*, Δ*espF*_*C*_, and *pespF*_*C*_. In contrast, the red fluorescent signals of Δ*espF, pespF*_*N*_, Δ*espF*_*N*_ were stronger. These groups were thus considered to have a weak ability in disrupting the mitochondrial Δψm.

The results of flow cytometry coincided with the above results. The cells of WT, *pespF, pespF*_*C*_, and Δ*espF*_*C*_ showed lower membrane potential than the Δ*espF, pespF*_*N*_, Δ*espF*_*N*_ groups. The findings of this experiment prove that *espF* effectively disrupts mitochondrial Δψm. The Δψm of Δ*espF*_*C*_ was low, whereas that of Δ*espF*_*N*_ was high, indicating that the EspF N-terminal domain plays an essential role in regulating mitochondrial apoptosis compared to the C-terminal domain.

After infection, different host cells were separated by flow cytometry (after double staining with annexin V-FITC and PI) based on the degree of apoptosis. The percentage of early and late apoptotic cells infected with WT, *pespF*, Δ*espF*_*C*_, and *pespF*_*C*_ was higher than that with Δ*espF*, Δ*espF*_*N*_, and *pespF*_*N*_. These results prove that *espF* plays a key role in accelerating apoptosis. The percentage of apoptotic cells infected with Δ*espF*_*C*_ was high, whereas that with Δ*espF*_*N*_ was significantly lower. And the caspase-3/9 activity of WT, *pespF* and Δ*espF*_*C*_ were enhanced, which was higher than Δ*espF*, Δ*espF*_*N*_ group. These findings indicate that the N-terminal of EspF plays a critical role in host cell apoptosis. Cell apoptosis contributes to the inflammation of intestinal epithelial cells, which consequently induces hemorrhagic colitis. Our findings were similar to that of Philippe et al., who reported that the N-terminal of EspF induces cell apoptosis. Furthermore, we have determined that the C-terminal of EspF alone does not induce cells to undergo apoptosis.

ROS are byproducts of aerobic respiration that are involved in signal transduction, immune responses, and regulation of gene expression and participate in the pathogenesis of various diseases and cancer (Shi et al., 1998; Vallyathan et al., 1998; Schieber and Chandel, 2014). Excessive ROS can destroy various cell components and induce lipid peroxidation of the cytomembrane. More importantly, these can induce DNA breakage, inhibit cell proliferation, and promote apoptosis (Jena, 2012; Dan Dunn et al., 2015). Our results showed that the WT, *pespF*_*C*_ and *pespF* generated more ROS than Δ*espF*, Δ*espF*_*N*_, and *pespF*_*N*_ within the host cells. These also disrupted oxygen metabolism and suppressed cell proliferation, which are hallmark features of apoptosis. On the other hand, the ROS of levels Δ*espF* and Δ*espF*_*N*_ significantly decreased. These findings reveal that the *espF* gene, particularly its N-terminal, plays a major role in the generation of ROS.

The EspF domains involved in tight junction disruption remain unclear (Dean et al., 2010). Our preliminary results show that N-terminal of the *espF* gene plays an important role in disrupting tight junctions of the host cell. Thereby allowing leakage of water and small molecules, which are symptoms of diarrhea. In the future, more research are needed to verify the its specific mechanism.

TNF-α is a diversified cytokine that causes various biological effects (Sethi et al., 2008). It mainly triggers three signal pathways, namely, apoptosis that is mediated by members of the caspase family, regulation of NF-κ B, which is mediated by adaptin TRAF, and activation of JNKMAPK. Our results showed that WT, *pespF*, and *espF*_*C*_ secrete more TNF-α than Δ*espF*, Δ*espF*_*N*_, *pespF*_*C*_, and *pespF*_*N*_ (*p* < 0.05). These results indicate that the deletion of the *espF* gene results in a decrease in TNF-α production. The N-terminal of EspF plays a critical role in this process. TNF-α activates members of the caspase family or the NF-κB signaling pathway, which in turn increases caspase-3/8 expression via TNFR1-TRADD-FADD and TNFR1-TRADD-TRAF (Hayden and Ghosh, 2014). In the present study, we verified that the reduction in caspase-3 expression could also be the consequence of secreting less TNF-α. Additional experiments exploring whether the N-terminal of EspF activates JNKMAPK are thus warranted.

Mice have always been the ideal animal model for studying EHEC infections (Wadolkowski et al., 1990; Mohawk and O'Brien, 2011). In this study, BALB/c mice were injected with mitomycin C (MMC) to increase their vulnerability to pathogenic bacteria (Al-Jumaili et al., 1992; Zhao et al., 2013). The highest mortality (50%) and colon weight (0.117 ± 0.014 g) was observed in the WT group. The first deaths and the earliest pathological symptoms were also observed in mice infected with WT. A lower number of BALB/c mice infected with Δ*espF* (30%) and Δ*espF*_*N*_ (20%) had died. However, the mortality rate (40%) and colon weight (0.110 ± 0.016 g) of mice infected with Δ*espF*_*C*_ was high and death occurred early.

The deletion of the N-terminal and the entire *espF* gene led to a significant reduction in mouse mortality and EHEC virulence, whereas the deletion of the C-terminal of EspF induced less severe effects. These findings indicate that the EspF N-terminal domain determines EHEC toxicity in the mouse model. The complementation strains were not involved in this experiment because 0.1% L-arabinose was required to induce the expression of the complementation gene (*espF*). L-arabinose was quantitatively added *in vitro* in this study. *In vivo*, some L-arabinose was absorbed by the BALB/c mice. Thus, its concentration could not be maintained at 0.1%, which in turn resulted in low levels of expression of complementation strains.

The findings of the present study prove that the C-terminal imparted minimal effects on cellular and mitochondrial apoptosis, inflammatory response, and animal toxicity. It also demonstrated that the C-terminal is not involved with the apoptotic effect of EspF and may play another cellular role. The C-terminal (73–248 aa) comprises four highly homologous proline-rich repeat (PRR) regions, each having one PxxP motif and one CRIB (Cdc42/Rac-interactive binding) domain (McNamara and Donnenberg, 1998). The src homology 3 (SH3) domain, which is 50–60 aa in length, mediates protein-protein interactions by binding to PRR regions (Mayer, 2001). For example, in the Src and Abl tyrosine kinase, the CRIB domain binds and activates N-WASP to form pedestals and cytoskeletal rearrangements via the Arp2/3-F-actin pathway (Alto et al., 2007). This presumably reflects that the C-terminal requires the N-terminal to perform its physiological function. A complete EspF protein is essential to its multifunctionality, and further research studies elucidating its role in apoptosis are warranted. In this experiment, the recovery effects of Δ*espF*_*N*_*/pespF*_*N*_ and Δ*espF*_*C*_*/pespF*_*C*_ were poor. This may because that these two complementation strains only express the peptide fragments of the N- or C-terminal. However, a complete structure is essential for EspF to play an efficient role.

We also evaluated the ability of Δ*espF* to induce A/E lesions. Lovo cells infected with WT, Δ*espF*, and *pespF* were stained with rhodamine-phalloidin and DAPI. The Lovo cells with A/E lesions showed intense staining and were counted (Figure S1). The results showed that the WT (35.03%) and *pespF* (32.27%) did not significantly differ in terms of the effects of Δ*espF* (26.42%). These findings also reveal that *espF* is not involved in the formation of A/E lesions, which coincide with the findings of previous studies (McNamara and Donnenberg, 1998; Shaw et al., 2005).

## Conclusions

EspF was injected into host cells using a type three secretion system (TTSS) after EHEC O157:H7 infection. EspF has multiple functions; its N-terminal promotes ROS generation, disrupts membrane potential of mitochondria and tight junction, increases the expression of inflammatory factor TNF-α, resulting in the cell apoptosis and death of mice. The findings of the present study provide information on the pathogenesis and molecular mechanisms of EHEC O157:H7.

## Author contributions

Conception or design of the work: XW and YD. Data collection: YH, MF, and CN. Data analysis and interpretation: BZ and WZ. Drafting the article: XW and YD. Critical revision of the article: CW. Final approval of the version to be published: QZ and CW.

### Conflict of interest statement

The authors declare that the research was conducted in the absence of any commercial or financial relationships that could be construed as a potential conflict of interest.

## Supplementary material

Figure S1 The espF isn't involved in the formation of AlE lesions. **(A)** Lovo cells were stained with rhodamine-phalloidin (actin) and DAPI (nuclei) after infection. The white arrows point to AlE lesions. **(B)** The proportion of Lovo cells which has formed AlE lesions (at least 3 slides, more than 50 cells per slide, ^**^*p* < 0.01 vs. other groups).

## References

[B1] Al-Jumaili I. Burke D. A. Scotland S. M. al-Mardini H. Record C. O. (1992). A method of enhancing verocytotoxin production by *Escherichia* coli. FEMS Microbiol. Lett. 72, 121–125. 10.1111/j.1574-6968.1992.tb05077.x 1505736

[B2] Alto N. M. Weflen A. W. Rardin M. J. Yarar D. Lazar C. S. Tonikian R.. (2007). The type III effector EspF coordinates membrane trafficking by the spatiotemporal activation of two eukaryotic signaling pathways. J. Cell Biol. 178, 1265–1278. 10.1083/jcb.200705021 17893247 PMC2064658

[B3] Chase-Topping M. Gally D. Low C. Matthews L. Woolhouse M. (2008). Super-shedding and the link between human infection and livestock carriage of *Escherichia coli* O157. Nat. Rev. Microbiol. 6, 904–912. 10.1038/nrmicro2029 19008890 PMC5844465

[B4] Dan Dunn J. Alvarez L. A. Zhang X. Soldati T. (2015). Reactive oxygen species and mitochondria: a nexus of cellular homeostasis. Redox Biol. 6, 472–485. 10.1016/j.redox.2015.09.005 26432659 PMC4596921

[B5] Dean P. Scott J. A. Knox A. A. Quitard S. Watkins N. J. Kenny B. (2010). The enteropathogenic *E*. coli effector EspF targets and disrupts the nucleolus by a process regulated by mitochondrial dysfunction. PLoS Pathog 6:e1000961. 10.1371/journal.ppat.1000961 20585567 PMC2891835

[B6] Frank C. Werber D. Cramer J. P. Askar M. Faber M. an der Heiden M.. (2011). Epidemic profile of Shiga-toxin-producing *Escherichia coli* O104:H4 outbreak in Germany. N. Engl. J. Med. 365, 1771–1780. 10.1056/NEJMoa1106483 21696328

[B7] Hayden M. S. Ghosh S. (2014). Regulation of NF-kappaB by TNF family cytokines. Semin. Immunol. 26, 253–266. 10.1016/j.smim.2014.05.004 24958609 PMC4156877

[B8] Heiman K. E. Mody R. K. Johnson S. D. Griffin P. M. Gould L. H. (2015). *Escherichia coli* O157 Outbreaks in the United States, 2003-2012. Emerg. Infect. Dis. 21, 1293–1301. 10.3201/eid2108.141364 26197993 PMC4517704

[B9] Holmes A. Muhlen S. Roe A. J. Dean P. (2010). The EspF effector, a bacterial pathogen's Swiss army knife. Infect. Immun. 78, 4445–4453. 10.1128/IAI.00635-10 20679436 PMC2976335

[B10] Honish L. Punja N. Nunn S. Nelson D. Hislop N. Gosselin G.. (2017). *Escherichia coli* O157:H7 Infections associated with contaminated pork products - Alberta, Canada, July-October 2014. MMWR Morb. Mortal. Wkly. Rep. 65, 1477–1481. 10.15585/mmwr.mm6552a5 28056011

[B11] Jena N. R. (2012). DNA damage by reactive species: mechanisms, mutation and repair. J. Biosci. 37, 503–517. 10.1007/s12038-012-9218-2 22750987

[B12] Kanayama A. Yahata Y. Arima Y. Takahashi T. Saitoh T. Kanou K.. (2015). Enterohemorrhagic *Escherichia coli* outbreaks related to childcare facilities in Japan, 2010-2013. BMC Infect. Dis. 15:539. 10.1186/s12879-015-1259-3 26589805 PMC4654900

[B13] Kaper J. B. Nataro J. P. Mobley H. L. (2004). Pathogenic *Escherichia coli*. Nat. Rev. Microbiol. 2, 123–140. 10.1038/nrmicro818 15040260

[B14] Li H. Jing H. Pang B. Zhao G. Yang J. Xu J. (2002). [Study on diarrhea disease caused by enterohemorrhagic *Escherichia coli* O157:H7 in Xuzhou city, Jiangsu province in 2000]. Zhonghua Liu Xing Bing Xue Za Zhi 23, 119–122. 10.3760/j.issn:0254-6450.2002.02.012 12015094

[B15] Mayer B. J. (2001). SH3 domains: complexity in moderation. J. Cell Sci. 114, 1253–1263. 11256992 10.1242/jcs.114.7.1253

[B16] McNamara B. P. Donnenberg M. S. (1998). A novel proline-rich protein, EspF, is secreted from enteropathogenic *Escherichia coli* via the type III export pathway. FEMS Microbiol. Lett. 166, 71–78. 10.1111/j.1574-6968.1998.tb13185.x 9741085

[B17] Michino H. Araki K. Minami S. Takaya S. Sakai N. Miyazaki M.. (1999). Massive outbreak of *Escherichia coli* O157:H7 infection in schoolchildren in Sakai City, Japan, associated with consumption of white radish sprouts. Am. J. Epidemiol. 150, 787–796. 10.1093/oxfordjournals.aje.a010082 10522649

[B18] Mohawk K. L. O'Brien A. D. (2011). Mouse models of *Escherichia coli* O157:H7 infection and shiga toxin injection. J. Biomed. Biotechnol. 2011:258185. 10.1155/2011/258185 21274267 PMC3022220

[B19] Moist L. Sontrop J. M. Garg A. X. Clark W. F. Suri R. S. Gratton R.. (2010). Risk of pregnancy-related hypertension within 5 years of exposure to drinking water contaminated with *Escherichia coli* O157:H7. J. Clin. Hypertens. (Greenwich). 12, 613–620. 10.1111/j.1751-7176.2010.00288.x 20695938 PMC8816438

[B20] Money P. Kelly A. F. Gould S. W. Denholm-Price J. Threlfall E. J. Fielder M. D. (2010). Cattle, weather and water: mapping *Escherichia coli* O157:H7 infections in humans in England and Scotland. Environ. Microbiol. 12, 2633–2644. 10.1111/j.1462-2920.2010.02293.x 20642796

[B21] Nagai T. Abe A. Sasakawa C. (2005). Targeting of enteropathogenic *Escherichia coli* EspF to host mitochondria is essential for bacterial pathogenesis: critical role of the 16th leucine residue in EspF. J. Biol. Chem. 280, 2998–3011. 10.1074/jbc.M411550200 15533930

[B22] Nougayrede J. P. Donnenberg M. S. (2004). Enteropathogenic *Escherichia coli* EspF is targeted to mitochondria and is required to initiate the mitochondrial death pathway. Cell Microbiol. 6, 1097–1111. 10.1111/j.1462-5822.2004.00421.x 15469437

[B23] Pennington H. (2010). *Escherichia coli* O157. Lancet 376, 1428–1435. 10.1016/S0140-6736(10)60963-4 20971366

[B24] Rangel J. M. Sparling P. H. Crowe C. Griffin P. M. Swerdlow D. L. (2005). Epidemiology of *Escherichia coli* O157:H7 outbreaks, United States, 1982-2002. Emerg. Infect. Dis. 11, 603–609. 10.3201/eid1104.040739 15829201 PMC3320345

[B25] Schieber M. Chandel N. S. (2014). ROS function in redox signaling and oxidative stress. Curr. Biol. 24, R453–R462. 10.1016/j.cub.2014.03.034 24845678 PMC4055301

[B26] Sethi G. Sung B. Aggarwal B. B. (2008). TNF: a master switch for inflammation to cancer. Front. Biosci. 13:5094–5107. 10.2741/3066 18508572

[B27] Shaw R. K. Cleary J. Murphy M. S. Frankel G. Knutton S. (2005). Interaction of enteropathogenic *Escherichia coli* with human intestinal mucosa: role of effector proteins in brush border remodeling and formation of attaching and effacing lesions. Infect. Immun. 73, 1243–1251. 10.1128/IAI.73.2.1243-1251.2005 15664974 PMC547083

[B28] Shi X. Castranova V. Halliwell B. Vallyathan V. (1998). Reactive oxygen species and silica-induced carcinogenesis. J. Toxicol. Environ. Heal. B. Crit. Rev. 1, 181–197. 10.1080/10937409809524551 9644327

[B29] Sinha K. Das J. Pal P. B. Sil P. C. (2013). Oxidative stress: the mitochondria-dependent and mitochondria-independent pathways of apoptosis. Arch. Toxicol. 87, 1157–1180. 10.1007/s00204-013-1034-4 23543009

[B30] Tahoun A. Siszler G. Spears K. McAteer S. Tree J. Paxton E.. (2011). Comparative analysis of EspF variants in inhibition of *Escherichia* coli phagocytosis by macrophages and inhibition of *E*. coli translocation through human- and bovine-derived M cells. Infect. Immun. 79, 4716–4729. 10.1128/IAI.00023-11 21875965 PMC3257939

[B31] Vallyathan V. Shi X. Castranova V. (1998). Reactive oxygen species: their relation to pneumoconiosis and carcinogenesis. Environ. Heal. Perspect. 106 (Suppl 5), 1151–1155. 10.1289/ehp.98106s51151 9788890 PMC1533374

[B32] Viswanathan V. K. Weflen A. Koutsouris A. Roxas J. L. Hecht G. (2008). Enteropathogenic E. coli-induced barrier function alteration is not a consequence of host cell apoptosis. Am. J. Physiol. Gastrointest Liver Physiol. 294, G1165–G1170. 10.1152/ajpgi.00596.2007 18356531 PMC3327053

[B33] Wadolkowski E. A. Burris J. A. O'Brien A. D. (1990). Mouse model for colonization and disease caused by enterohemorrhagic *Escherichia* coli O157:H7. Infect. Immun. 58, 2438–2445. 2196227 10.1128/iai.58.8.2438-2445.1990 PMC258838

[B34] Zhao S. Zhou Y. Wang C. Yang Y. Wu X. Wei Y.. (2013). The N-terminal domain of EspF induces host cell apoptosis after infection with enterohaemorrhagic *Escherichia coli* O157:H7. PLoS ONE 8:e55164. 10.1371/journal.pone.0055164 23372831 PMC3555930

