# Supplementary figures and images for: The EspF N-Terminal of Enterohemorrhagic Escherichia coli O157:H7 EDL933w Imparts Stronger Toxicity Effects on HT-29 Cells than the C-Terminal
Source: Front Cell Infect Microbiol. 2017 Sep 21;7:410. doi: 10.3389/fcimb.2017.00410 (PMC5613159; doi:10.3389/fcimb.2017.00410)

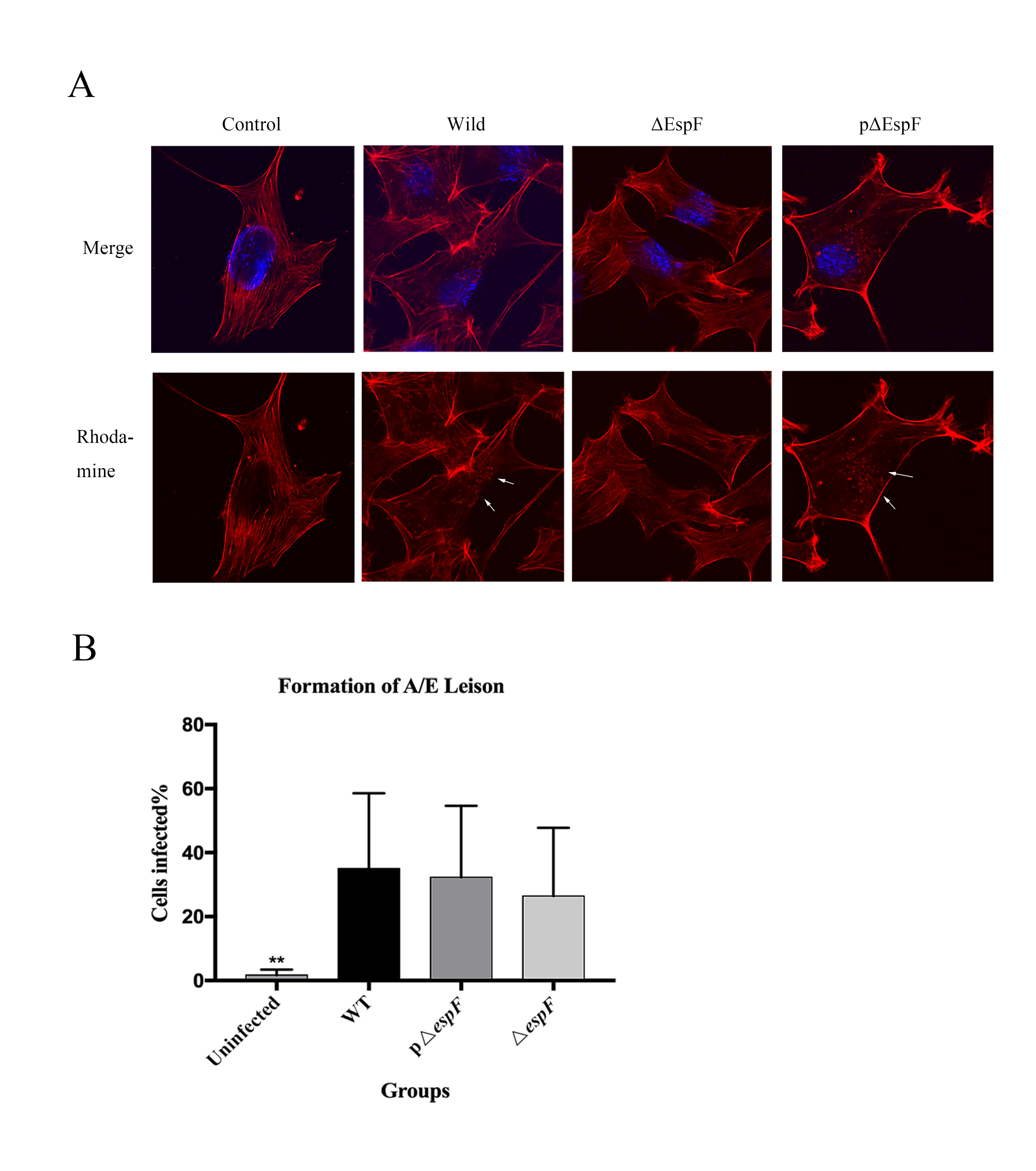

Supplement: Figure S1 — The espF isn't involved in the formation of AlE lesions. (A) Lovo cells were stained with rhodamine-phalloidin (actin) and DAPI (nuclei) after infection. The white arrows point to AlE lesions. (B) The proportion of Lovo cells which has formed AlE lesions (at least 3 slides, more than 50 cells per slide, **p < 0.01 vs. other groups). [file Image1.tif]
